# Associations Between Physical Activity and Nutrition Self-efficacy and Self-Reported Physical Activity in Residents of Resource-limited Neighborhoods

**DOI:** 10.1016/j.focus.2026.100476

**Published:** 2026-01-09

**Authors:** Foster Osei Baah, Colby Ayers, Lola R. Ortiz-Whittingham, Valerie M. Mitchell, Billy S. Collins, Marcus R. Andrews, Marie Marah, Frederick Ayensu, Kingsley Boakye, Adwoa Gyamfi, Katherine J. Tolentino, Gwenyth R. Wallen, Jacob K. Karuiki, Yvonne Baumer, Tiffany M. Powell-Wiley

**Affiliations:** 1 Nell Hodgson Woodruff School of Nursing, Emory University, Atlanta, Georgia; 2 UT Southwestern Medical Center, Dallas, Texas; 3 Social Determinants of Obesity and Cardiovascular Risk Laboratory, National Heart Lung and Blood Institute, NIH, Bethesda, Maryland; 4 HopeXchange Medical Center, Kumasi, Ghana; 5 Julius Center for Health Sciences and Primary Care, University Medical Center, Utrecht University, Utrecht, The Netherlands; 6 School of Nursing, Kwame Nkrumah University of Science and Technology, Kumasi, Ghana; 7 Clinical Center, NIH, Bethesda, Maryland; 8 Intramural Research Program, National Institute on Minority Health and Health Disparities, NIH, Bethesda, Maryland

**Keywords:** Health behaviors, physical activity, obesity, self-efficacy

## Abstract

**Introduction:**

The authors explored the relationship between lifestyle behavior, self-efficacy, and self-reported physical activity among individuals with overweight/obesity residing in resource-limited neighborhoods.

**Methods:**

Using data from the Washington, DC Cardiovascular Health and Needs Assessment and the Step It Up digital health intervention study, the authors examined the relationships between self-efficacy and self-reported physical activity participation. Individuals completed the Physical Activity and Nutrition Self-Efficacy scale and the Global Physical Activity Questionnaire, which assesses self-reported physical activity participation. Logistic and gamma regression models were performed to assess the associations between Physical Activity and Nutrition Self-Efficacy quintiles and (1) the odds of any physical activity participation and (2) the duration of physical activity participation.

**Results:**

The sample (N=227) had a mean age of 57.71 (±12.60) years and BMI of 34.55 (±7.19) and were predominantly female (90.75%). The majority had college education (57.55%) and less than $60,000 annual income (44.15%). Individuals who scored in the third quintile were 3.26 (95% CI=1.18, 9.02) and 3.97 (95% CI=1.46, 10.82) times more likely to report moderate physical activity and muscle-strengthening activity participation, respectively, than those who scored in the first quintile, after adjusting for sociodemographic factors. Those who scored in the third and fifth quintiles were 3.91 (95% CI=1.28, 11.97) and 5.92 (95% CI=1.90,18.47) times more likely to meet the guideline-recommended 150 minutes weekly moderate-to-vigorous physical activity.

**Conclusions:**

Higher physical activity self-efficacy is associated with higher odds of participation, physical activity duration, and the odds of meeting the guideline-recommended weekly moderate-to-vigorous physical activity. Interventions that augment self-efficacy may be particularly important in improving cardiovascular health by enhancing physical activity in individuals with obesity.

## Highlights

• Higher self-efficacy is associated with higher odds of physical activity participation.
• Higher self-efficacy is also associated with higher mean level leisure-time moderate physical activity when participation occurs than lower self-efficacy.
• Augmenting self-efficacy may improve cardiovascular health by enhancing physical activity in community-dwelling individuals with obesity residing in resource-limited neighborhoods.

## INTRODUCTION

Social determinants of health reflect social and environmental factors that shape the lived experiences of population groups, define the systems put in place to deal with illness, and affect associated cardiovascular health behaviors that subsequently influence variations in disease risk, development, and management.^1^^,^^2^ Cardiovascular health behaviors include self-care (individual-level treatment adherence [maintenance], symptom monitoring/recognition [symptom perception], and actions used to manage symptoms [management]),^3^, ^4^, ^5^ physical activity (PA), and dietary choices that promote cardiovascular health. Community-dwelling individuals perform these health behaviors within the context of their neighborhoods and individual social position shaped by social drivers of health.

Self-efficacy, a construct from Bandura’s social cognitive theory, refers to an individual’s capability or confidence in performing specific behaviors and is believed to affect one’s choices and persistence in performing health-enhancing behaviors within a specific social and neighborhood context.^6^ Increased self-efficacy is known to be associated with reduced sedentary behaviors^7^ and better health-related quality of life.^8^^,^^9^ For instance, increased self-efficacy is associated with improved self-care behaviors such as medication adherence in patients with hypertension.^10^ Self-efficacy has also been shown to moderate the relationship between PA changes and perceived social support in Black and White high-school girls (girls with a strong social support had less of a decline in PA if they had increased self-efficacy) in South Carolina.^9^^,^^11^ Yet, investigators remain unclear about the relationship between self-efficacy and self-reported PA among population groups with overweight or obesity, particularly among underrepresented individuals residing in resource-limited neighborhoods. It is imperative to better understand the relationship between an individual’s self-efficacy to participate in healthy behavior choices that define disease risk and the lifestyle behaviors within their neighborhood context to identify targets for feasible and sustainable behavioral interventions to improve population health.

Therefore, the aim of this study was to explore the relationship between Physical Activity and Nutrition Self-Efficacy (PANSE) and specific self-reported PA types among residents with overweight or obesity in resource-limited Washington, DC neighborhoods by addressing 2 specific questions of interest: (1) what is the relationship between PANSE and the probability of participating in aerobic physical activities (leisure-time moderate PA [MPA] and leisure-time vigorous PA [VPA] and muscle-strengthening activity [MSA]) and (2) what is the relationship between PANSE and the duration the individual spent participating in MPA, VPA, and MSA when participation occurred?. The authors also explored the probability of meeting the guideline-recommended 150 minutes per week of moderate–vigorous PA (MVPA). The authors hypothesized the following: (1) higher PANSE score quintiles will be associated with increased odds of participation in MPA, VPA, MSA, and meeting the guideline-recommended MVPA per week than the first quintile and (2) when PA participation occurred, higher PANSE quintiles will be associated with increased time spent participating in the PA types than the first quintile.

## METHODS

### Study Participants

This is a secondary analysis of combined data from the Washington, DC Cardiovascular Health and Needs Assessment and the Step It Up Digital Health, Community Engaged Physical Activity Intervention. The methods and procedures of both parent studies have been reported elsewhere.^12^, ^13^, ^14^, ^15^ Briefly, the DC Cardiovascular Health and Needs Assessment is a community-based participatory observational study that evaluated the cardiovascular health and psychosocial factors, cultural norms, and neighborhood characteristics among individuals living in resource-limited neighborhoods in Washington, DC (Wards 5,7, and 8).^13^^,^^14^ Compared with other neighborhoods in the metropolitan area, these 3 wards have largely Black residents with lower median household income and the highest cardiovascular disease burden in the city. Community-dwelling adults (*n*=119) who were aged 19–85 years, spoke and wrote English at an 8^th^ grade level, and had internet connectivity were enrolled from 4 churches within the 3 wards. Step It Up is a community-engaged and place-tailored digital health intervention that is evaluating the impact of neighborhood-tailored messaging on PA and cardiovascular outcomes and the role of multilevel environmental and psychosocial factors on PA engagement in the same resource-limited Washington, DC neighborhoods (Wards 5,7, and 8) and surrounding Prince George’s County, Maryland.^12^ Step It Up participants (*n*=108) were community-dwelling African American women (aged 21–75 years) who had smart phones, spoke and wrote English proficiently, and had overweight or obesity (BMI ≥25 kg/m^2^). Women in the second and third trimesters of pregnancy were excluded in both parent studies, and participant’s medical history, cardiovascular disease risk, and health behavior were collected. All participants in both parent studies provided informed consent, and IRB approval was obtained from the National Heart, Lung, and Blood (Protocols 17-H-0162 and 13-H-0183). The analytic sample (N=227) included only participants with data across the main predictor and outcome variables of interest from the parent studies.

### Measures

All participants completed the PANSE scale, a validated 11-item 9-point Likert scale that measures the participants’ degree of confidence in their capability to participate in PA and dietary behaviors related to weight loss.^16^ It is composed of 2 knowledge domains (PA [3 items] and dietary [8 items]) that influence weight loss. The range of total scores is 11–99, with higher scores reflecting higher self-efficacy and a Cronbach alpha of 0.89.^16^ The authors created PANSE score quintile ranks, with the first and fifth quintiles indicating the lowest and highest 20% of PANSE scores, respectively.

The amount of time that individuals spent participating in leisure-time MPA, VPA, weighted MVPA, and MSA was calculated using the Global Physical Activity Questionnaire for self-reported PA.^17^ The Global Physical Activity Questionnaire measures self-reported time spent in specific types of PA by adults and has demonstrated adequate test–retest reliability (Cronbach’s alpha <0.8).^18^ The authors calculated MPA as leisure-time MPA and VPA as leisure-time VPA per week. The authors then calculated weighted MVPA as the sum of minutes of MPA per week and double the minutes of VPA per week. MSA was also calculated as the number of times per week that the individual participated in MSA.

### Statistical Analysis

Data description was done using means with SDs and percentages for continuous and categorical variables, respectively. The authors used 2-part modeling to address (1) the probability that a person *j* did not participate in a specific PA type on a specific day *i* and (2) how much of that activity occurred when the individual participated in it.^19^ That is, the authors used a multilevel logistic regression to predict participation in a specific PA type (log odds of activity) and a multilevel gamma regression to predict the duration of the PA type when the individual participated in that PA type as a function of PANSE and the sociodemographic variables (age, sex [self-reported male or female], income, and education).^19^ Hence, for each PA type, the authors created 2-part models (zero and positive portions) to address the study aims. The authors created 3 two-part models for each of the PA types: (1) unadjusted model, (2) Model 1 (adjusted for age and sex), and (3) Model 2 (including all the covariates in Model 1 in addition to participants’ education and income). For MVPA, the authors only modeled the odds of meeting the guideline-recommended 150 minutes per week of MVPA instead of the odds of participation and duration of participation. All data analysis were done using SAS 9.4.^20^

## RESULTS

Table 1 describes the sociodemographic, clinical characteristics, and variables of interest, with continuous variables summarized by their means with their SDs and categorical variables presented as counts with their percentages. The sample (N=227) were predominantly Black/African American (99.12%) females (90.75%) with college education (57.55%) and less than $60,000 annual income (44.15%) with a mean age of 57.71 (±12.60) years and BMI of 34.55 (±7.19) kg/m^2^. The mean systolic and diastolic blood pressures were 132.33 (±4.76) mmHg and 77.63 (±11.50) mmHg, respectively. All the estimates for the logistic and gamma regressions are reported in Appendix Tables 1–4 (available online).

**Table 1:** Sociodemographic and Clinical Characteristics (N=227)

Participant characteristics	*n*	Summary Mean±SD or counts (%)
Age, years	226	57.71±12.60
Weight, kg	204	93.37 (±20.40)
Waist circumference, cm	206	102.83 (±16.17)
Hip circumference, cm	206	118.10±15.81
BMI, kg/m^2^	206	34.55±7.19
Systolic blood pressure, mmHg	204	132.33 (±14.76)
Diastolic blood pressure, mmHg	204	77.63 (±11.50)
PANSE	223	73.96±15.88
Sex	227	
Female		206 (90.75%)
Male		21 (9.25%)
Race	227	
Black or African American		225 (99.12%)
Native Hawaiian or other Pacific Islander		1 (0.44%)
White		1 (0.44%)
Education	212	
College/graduate degree		122 (57.55%)
Some college/technical degree		61 (28.77%)
High school or less		29 (13.68%)
Annual income	188	
≥$100,000		44 (23.40%)
$60,000–$99,999		61 (32.45%)
<$60,000		83 (44.15%)
Physical activity variables		Median (25th, 75th)
Moderate leisure, minutes/week	225	60.00 (0.00, 180.00)
Vigorous leisure, minutes/week	225	0.00 (0.00, 60.00)
Weighted leisure-time moderate to vigorous, minutes/week	181	120 (1,450)
Muscle-strengthening activity, number of times/week	225	1.00 (0.00, 3.00)

The odds of participating in leisure-time MPA were 3.50 (95% CI=1.40, 8.73) times higher among participants with PANSE scores in the third quintile than among those with scores in the first quintile in the unadjusted model (Table 2). The odds of participating in leisure-time MPA remained 3.26 (95% CI=1.18, 9.02) times higher for those in the third than for those in the first quintile in the final model (fully adjusted for age, sex, education, and income). The odds of MPA participation for those with PANSE scores in the other quintiles relative to the odds for those in the first quintile were not statistically significant. When individuals participated in leisure-time MPA (unadjusted gamma model), having PANSE scores in the fifth relative to the first quintile was associated with 2.53 (95% CI=1.46, 4.36) times higher mean leisure-time MPA. In the fully adjusted model (Model 2), having PANSE scores in the fourth and fifth quintiles relative to having them in the first quintile was significantly associated with 2.17 (95% CI=1.19, 3.96) and 2.88 (95% CI=1.65, 5.01) times higher mean leisure-time MPA (Table 2).

**Table 2:** Leisure-Time Moderate Physical Activity: ORs and Mean Ratios

	Unadjusted		Model 1		Model 2	
Parameter	OR	LCL, UCL	OR	LCL, UCL	OR	LCL, UCL
ORs and CIs from logistic regression estimates						
Q2	1.08	0.46, 2.56	1.12	0.47, 9.88	0.87	0.32, 2.34
Q3	3.50	1.40, 8.73	3.86	1.51, 9.88	3.26	1.18, 9.02
Q4	2.03	0.85, 4.82	2.09	0.87, 5.00	1.32	0.49, 3.58
Q5	1.99	0.83, 4.79	2.12	0.87, 5.17	1.83	0.70, 4.78
Age	—	—	1.02	0.10, 1.04	1.02	0.99, 1.05
Male	—	—	1.34	0.49, 3.67	1.87	0.60, 5.85
College	—	—	—	—	2.71	0.97, 7.57
Some college	—	—	—	—	2.03	0.68, 6.12
High income	—	—	—	—	1.48	0.63, 3.43
Low income	—	—	—	—	1.79	0.85, 3.76
Mean ratios and CIs from gamma regression estimates						
Q2	1.57	0.89, 2.79	1.63	0.91, 2.93	1.37	0.74, 2.53
Q3	1.58	0.93, 2.66	1.65	0.96, 2.84	1.52	0.88, 2.62
Q4	1.65	0.97, 2.83	1.72	0.10, 2.97	2.17	1.19, 3.96
Q5	2.53	1.46, 4.36	2.60	1.50, 4.50	2.88	1.65, 5.01
Age	—	—	1.01	0.99, 1.02	1.01	0.99, 1.03
Male	—	—	1.04	0.61, 1.79	1.18	0.67, 2.08
College	—	—	—	—	1.31	0.69, 2.50
Some college	—	—	—	—	1.41	0.70, 2.82
High income	—	—	—	—	1.35	0.86, 2.12
Low income	—	—	—	—	1.27	0.84, 1.93

LCL, lower confidence limit; Q, quartile; UCL, upper confidence limit.

None of the logistic models were statistically significant for leisure-time VPA (Appendix Table 2, available online). The third quintile relative to the first was significantly associated with 3.44 (95% CI=1.33, 8.91) and 4.03 (95% CI=1.60, 10.15) times higher mean leisure-time VPA in the unadjusted model and Model 1 (age and sex adjusted) of the gamma model (Table 3). However, the fully adjusted gamma model was not statistically significant.

**Table 3:** Leisure-Time Vigorous Physical Activity: ORs and Mean Ratios

	Unadjusted		Model 1		Model 2	
Parameter	OR	LCL, UCL	OR	LCL, UCL	OR	LCL, UCL
ORs and CIs from logistic regression estimates						
Q2	1.08	0.46, 2.56	1.12	0.47, 9.88	0.87	0.32, 2.34
Q3	3.50	1.40, 8.73	3.86	1.51, 9.88	3.26	1.18, 9.02
Q4	2.03	0.85, 4.82	2.10	0.87, 5.00	1.32	0.49, 3.58
Q5	1.99	0.83, 4.79	2.12	0.87, 5.17	1.83	0.70, 4.78
Age	—	—	1.02	0.10, 1.04	1.02	0.99, 1.05
Male	—	—	1.34	0.49, 3.67	1.87	0.60, 5.85
College	—	—	—	—	2.71	0.97, 7.57
Some college	—	—	—	—	2.03	0.68, 6.12
High income	—	—	—	—	1.48	0.64, 3.43
Low income	—	—	—	—	1.79	0.85, 3.76
Mean ratios and CIs from gamma regression estimates						
Q2	1.31	0.50, 3.44	1.68	0.64, 4.40	1.11	0.42, 2.95
Q3	3.44	1.33, 8.91	4.03	1.60, 10.15	2.21	0.88, 5.53
Q4	1.62	0.57, 4.57	1.93	0.70, 5.31	0.78	0.28, 2.18
Q5	1.27	0.50, 3.25	1.54	0.61, 3.89	1.19	0.50, 2.83
Age	—	—	1.03	1.00, 1.05	1.01	0.99, 1.04
Male	—	—	0.61	0.24, 1.56	0.55	0.22, 1.38
College	—	—	—	—	3.52	1.45, 8.59
Some college	—	—	—	—	5.66	1.96, 16.38
High income	—	—	—	—	0.40	0.20, 0.78
Low income	—	—	—	—	0.52	0.25, 1.11

LCL, lower confidence limit; Q, quartile; UCL, upper confidence limit.

Participants with PANSE scores in the third and fifth quintiles relative to those in the first quintile were significantly associated with 3.91 (95% CI=1.28, 11.97) and 5.92 (95% CI=1.90, 18.47) times higher odds of meeting the guideline-recommended 150-minute weekly MVPA on the basis of the unadjusted model (Table 4). In Model 2 (fully adjusted), having PANSE scores in the fifth compared with having them in the first quintile was significantly associated with 4.75 (95% CI=1.46, 15.45) times higher odds of meeting the guideline-recommended 150-minute weekly MVPA.

**Table 4:** Weighted Leisure-Time Moderate-to-Vigorous Physical Activity: ORs of Meeting the Guideline-Recommended 150 Minutes per Week Moderate-to-Vigorous Physical Activity

	Unadjusted		Model 1		Model 2	
Parameter	OR	LCL, UCL	OR	LCL, UCL	OR	LCL, UCL
ORs and CIs from logistic regression estimates						
Q2	2.29	0.77, 6.83	2.33	0.77, 6.99	2.31	0.72, 7.36
Q3	3.91	1.28, 11.97	3.83	1.24, 11.80	2.93	0.91, 9.43
Q4	2.33	0.77, 7.05	2.41	0.79, 7.34	2.15	0.64, 7.20
Q5	5.92	1.90, 18.47	5.93	1.89, 18.57	4.75	1.46, 15.45
Age	—	—	0.99	0.96, 1.01	0.10	0.97, 1.02
Male	—	—	1.06	0.38, 2.93	1.01	0.33, 3.07
College	—	—	—	—	2.43	0.68, 8.61
Some college	—	—	—	—	1.98	0.51, 7.74
High income	—	—	—	—	1.34	0.57, 3.18
Low income	—	—	—	—	1.40	0.63, 3.11

LCL, lower confidence limit; Q, quartile; UCL, upper confidence limit.

Participants with PANSE scores in the third and fifth quintiles were significantly associated with 2.99 (95% CI=1.21, 7.34) and 3.45 (95% CI=1.37, 8.66) times higher odds of participating in MSA, respectively, than those with scores in the first quintile on the basis of the unadjusted model (Table 5). These higher odds for the third and fifth PANSE quintiles remained statistically significant in the fully adjusted model relative to the first quintile with 3.97 (95% CI=1.46, 10.82) and 4.24 (95% CI=1.56,11.50) respective higher odds of participating in MSA. However, all the gamma models for MSA were not statistically significant.

**Table 5:** Muscle-Strengthening Activity: ORs and Mean Ratios

Parameter	Unadjusted		Model 1		Model 2	
	OR	LCL, UCL	OR	LCL, UCL	OR	LCL, UCL
ORs and CIs from logistic regression estimates						
	OR	LCI, UCI	OR	LCI, UCI	OR	LCI, UCI
Q2	1.98	0.83, 4.70	1.93	0.81, 4.62	2.09	0.78, 5.57
Q3	2.99	1.21, 7.34	2.93	1.18, 7.28	3.97	1.46, 10.82
Q4	1.75	0.73, 4.21	1.74	0.72, 4.17	1.82	0.66, 5.00
Q5	3.45	1.37, 8.66	3.42	1.36, 8.62	4.24	1.56, 11.50
Age	—	—	1.00	0.98, 1.02	1.00	0.98, 1.03
Male	—	—	1.25	0.48, 3.22	1.38	0.48, 3.96
College	—	—	—	—	1.10	0.39, 3.08
Some college	—	—	—	—	0.98	0.32, 3.01
High income	—	—	—	—	1.24	0.55, 2.79
Low income	—	—	—	—	1.50	0.72, 3.14
Mean ratios and CIs from gamma regression estimates						
Q2	0.89	0.60, 1.33	0.87	0.58, 1.31	0.98	0.63, 1.53
Q3	1.03	0.70, 1.54	1.01	0.67, 1.51	1.30	0.85, 1.99
Q4	0.91	0.61, 1.38	0.89	0.59, 1.35	1.08	0.68, 1.71
Q5	1.20	0.81, 1.78	1.20	0.81, 1.78	1.35	0.89, 2.05
Age	—	—	1.00	0.99, 1.01	1.01	1.00, 1.02
Male	—	—	1.30	0.90, 1.88	0.93	0.63, 1.38
College	—	—	—	—	1.10	0.74, 1.64
Some college	—	—	—	—	1.50	0.97, 2.31
High income	—	—	—	—	1.51	1.10, 2.06
Low income	—	—	—	—	0.82	0.62, 1.09

LCL, lower confidence limit; Q, quartile; UCL, upper confidence limit.

## DISCUSSION

The authors explored the associations between PANSE and the probability of self-reported leisure-time MPA, VPA, meeting the guideline-recommended 150 minutes of MVPA per week, and MSA among community-dwelling individuals with overweight or obesity and residing in resource-limited Washington, DC neighborhoods. Generally, the authors found increased odds of participation in leisure-time MPA and MSA and meeting the recommended MVPA guidelines in individuals within higher PANSE quintiles. Higher PANSE quintiles were also associated with increased time spent in leisure-time MPA but not VPA and MSA. Prior studies have demonstrated that self-efficacy is a modifiable factor for targeted intervention to improve PA^21^^,^^22^ and is a predictor of PA engagement in minoritized populations.^23^, ^24^, ^25^, ^26^, ^27^, ^28^, ^29^ The findings are consistent with those of these prior studies and add to the existing literature by demonstrating that among individuals with overweight or obesity living in resource-limited neighborhoods, higher self-efficacy is associated not only with increased odds of leisure-time PA engagement and almost threefold higher mean leisure-time MPA when participation occurs but also with over fourfold higher odds of meeting the guideline-recommended MVPA per week in this small cohort.

The impact of PA on health is profound, with consistent PA known to have cardioprotective effects even in the absence of weight loss in individuals with obesity.^30^, ^31^, ^32^, ^33^ However, several barriers can impede PA, including low PA self-efficacy and weight-related stigma,^34^, ^35^, ^36^ with 86% of adults not meeting the guideline-recommended 150 minutes per week of MVPA and 2 or more days of MSA.^37^ There is also increased PA intervention dropout among individuals with obesity,^38^ and those who go through the course of the intervention identify a lack of self-motivation, time constraints, and family obligations as barriers to sustained PA participation over time.^39^ This evidence, taken together with the findings, underscores the imperative to identify intervention targets that enhance PA-related self-efficacy to improve both PA engagement and the amount of time spent in PA when participation occurs.

Although self-efficacy may be a major target for PA engagement and adherence, other psychosocial factors may also influence PA engagement. For instance, social support (family and friends and perceived PA benefits) interacts with both self-efficacy and neighborhood environment characteristics (walkability and esthetics) to influence PA engagement in community-dwelling adults.^40^, ^41^, ^42^, ^43^, ^44^ Stronger motivation, perceived health, and a sense of belonging in the community have also been associated with increased PA participation.^45^, ^46^, ^47^ This evidence together with the findings suggests that future interventions aimed at getting community-dwelling individuals to the recommended PA guidelines should be multilevel, thus addressing policy-, neighborhood-, and individual-level barriers to PA engagement.^1^^,^^2^

PA as a health behavior has emerged as a pivotal component of disease prevention and disease maintenance globally, with increasing evidence suggesting that 4–12 minutes of vigorous-intensity PA per day reallocated from less vigorous PA has cardioprotective effects such as reduced BMI and lower HbA1c.^48^^,^^49^ However, U.S. adults are behind on the PA objectives of the *Healthy People 2030*, with just 25% of U.S. adults meeting the recommended aerobic and muscle-strengthening guidelines.^50^ This underscores the need to examine self-efficacy, personal urgency, and capabilities of underserved community-dwelling individuals with chronic cardiometabolic-related diseases such as cardiovascular–kidney–metabolic syndrome and their risk factors (Life’s Essential 8).^51^ A clearer understanding of the impact of these intrinsic factors and their mechanistic role in health behaviors (e.g., PA, diet, medication adherence, and others) coupled with the individuals’ neighborhood context will identify targets for sustainable multilevel (individual-, community-, and policy-level) interventions.

### Limitations

Although this study’s findings contribute to the literature on self-efficacy and PA, the authors recognize limitations related to using secondary data and a cross-sectional approach. The study population was predominantly African American and urban dwelling, which limits generalizability of the study findings to other populations. However, data from African American populations in resource-limited neighborhoods can aid in understanding potential intervention targets and associated implementation barriers for a population with high rates of physical inactivity and high risk for cardiovascular disease. The authors also used self-reported PA data, which may be associated with recall and response bias. However, the parent study employed a well-conceptualized study design and validated self-report measures for data collection.

## CONCLUSIONS

PA self-efficacy is associated with PA engagement and the amount of time spent in PA when participation occurs. Self-efficacy is an intrinsic factor that moderates health-enhancing behaviors in individuals with chronic illness. PA as a health behavior is shaped by neighborhood and the socioeconomic context that limits or enhances the self-care choices of populations in resource-limited neighborhoods. Perhaps, interventions that augment self-efficacy may be particularly important in improving cardiovascular health by enhancing PA in African Americans with obesity residing in resource-limited neighborhoods. Future interventional studies addressing PA in populations in resource-limited neighborhoods should consider targeting self-efficacy in conjunction with other external factors that impede PA.

## Appendix Supplementary materials

Image, application 1

## Footnotes

Supplementary material associated with this article can be found in the online version at doi:10.1016/j.focus.2026.100476.
