# Supplementary material for: Associations Between Physical Activity and Nutrition Self-efficacy and Self-Reported Physical Activity in Residents of Resource-limited Neighborhoods
Source: AJPM Focus. 2026 Jan 9;5(4):100476. doi: 10.1016/j.focus.2026.100476 (PMC13195610; doi:10.1016/j.focus.2026.100476)
Supplement: Supplementary file 1 [file mmc1.docx]

**Appendix Tables**

**Appendix Table 1: Estimates for Logistic and Gamma models: Moderate Leisure-time Physical Activity**

| Parameter | Unadjusted | | Model 1 | | Model 2 | |
| --- | --- | --- | --- | --- | --- | --- |
| **Multilevel logistic regression** | | | | | | |
|  | Estimate (SE) | P Value | Estimate (SE) | P Value | Estimate (SE) | P-Value |
| Intercept | -0.3023 (0.3198) | 0.3446 | -1.3807 (0.7662) | 0.0715 | -2.3964 (1.0287) | 0.0198* |
| PANSE (Ref: Q1) |  | | | | | |
| Q2 | 0.0791(0.4385) | 0.8568 | 0.1126 (0.4459) | 0.8007 | -0.1383 (0.5042) | 0.7839 |
| Q3 | 1.2514 (0.4668) | 0.0073** | 1.3510 (0.4791) | 0.0048** | 1.1817 (0.5194) | 0.0229* |
| Q4 | 0.7077 (0.4415) | 0.1089 | 0.7376 (0.4450) | 0.0974 | 0.2785 (0.5082) | 0.5837 |
| Q5 | 0.6879 (0.4485) | 0.1250 | 0.7505 (0.4548) | 0.0989 | 0.6065 (0.4890) | 0.2149 |
| Age | - | - | 0.0176 (0.0115) | 0.1244 | 0.0185 (0.0134) | 0.1682 |
| Male | - | - | 0.2943 (0.5130) | 0.5661 | 0.6272 (0.5810) | 0.2803 |
| College | - | - | - | - | 0.9981 (0.5235) | 0.0566 |
| Some College | - | - | - | - | 0.7099 (0.5619) | 0.2064 |
| High Income | - | - | - | - | 0.3899 (0.4295) | 0.3640 |
| Low Income | - | - | - | - | 0.5820 (0.3794) | 0.1251 |
| Fit Indices |  | | | | | |
| AIC | 294.555 | | 295.744 | | 251.961 | |
| -2 Log L | 284.555 | | 281.744 | | 229.961 | |
| **Multilevel gamma regression** | | | | | | |
| Intercept | 4.9624 (0.2149) | <.0001*** | 4.5664 (0.5169) | <.0001*** | 3.8543 (0.6520) | <.0001*** |
| PANSE |  | | | | | |
| Q2 | 0.4537 (0.2924) | 0.1207 | 0.4906 (0.2987) | 0.1005 | 0.3117 (0.3140) | 0.3209 |
| Q3 | 0.4558 (0.2675) | 0.0883 | 0.5016 (0.2769) | 0.0700 | 0.4171 (0.2787) | 0.1345 |
| Q4 | 0.5025 (0.2744) | 0.0671 | 0.5430 (0.2779) | 0.0507 | 0.7746 (0.3071) | 0.0117** |
| Q5 | 0.9264 (0.2786) | 0.0009*** | 0.9541 (0.2806) | 0.0007*** | 1.0564 (0.2829) | 0.0002*** |
| Age | - | - | 0.0061 (0.0073) | 0.4039 | 0.0102 (0.0082) | 0.2155 |
| Male | - | - | 0.0388 (0.2759) | 0.8883 | 0.1628 (0.2896) | 0.5741 |
| College | - | - | - | - | 0.2735 (0.3287) | 0.4054 |
| Some Colleges | - | - | - | - | 0.3423 (0.3542) | 0.3339 |
| High Income | - | - | - | - | 0.2988 (0.2321) | 0.1980 |
| Low Income | - | - | - | - | 0.2397 (0.2133) | 0.2611 |
| **Fit Indices** | | | | | | |
| AIC | 1558.7899 | | 1562.0877 | | 1276.7089 | |
| BIC | 1575.5149 | | 1584.3877 | | 1307.7285 | |

**Appendix Table 2: Estimates for Logistic and Gamma models: Leisure time Vigorous Physical Activity**

| Parameter | Unadjusted | | Model 1 | | Model 2 | |
| --- | --- | --- | --- | --- | --- | --- |
| **Multilevel logistic regression** | | | | | | |
|  | Estimate (SE) | P Value | Estimate (SE) | P Value | Estimate (SE) | P-Value |
| Intercept | -1.7346 (0.4428) | <.0001*** | -1.3623 (0.8644) | 0.1150 | -2.1426(1.1284) | 0.0576 |
| PANSE (Ref: Q1) |  | | | | | |
| Q2 | 0.6931 (0.5557) | 0.2123 | 0.6865 (0.5595) | 0.2199 | 0.5281 (0.5978) | 0.3770 |
| Q3 | 0.9323 (0.5545) | 0.0927 | 0.9099 (0.5592) | 0.1037 | 0.8106 (0.5856) | 0.1662 |
| Q4 | 0.2031 (0.5900) | 0.7306 | 0.2030 (0.5907) | 0.7311 | 0.0612 (0.6564) | 0.9257 |
| Q5 | 1.0415 (0.5507) | 0.0586 | 1.0265 (0.5521) | 0.0630 | 0.9539 (0.5774) | 0.0985 |
| Age | - | - | -0.00619 (0.0126) | 0.6234 | 0.00131 (0.0144) | 0.9277 |
| Male | - | - | -0.0962 (0.5486) | 0.8608 | 0.0741 (0.5844) | 0.8991 |
| College | - | - | - | - | -0.1213 (0.6048) | 0.8410 |
| Some College | - | - | - | - | -0.0405 (0.6425) | 0.9497 |
| High Income | - | -- | - | - | 1.1052 (0.4751) | 0.0200* |
| Low Income | - | - | - | - | 0.9169 (0.4355) | 0.0353* |
| Fit Indices |  | | | | | |
| AIC | 242.142 | | 242.142 | | 217.758 | |
| -2 Log L | 240.142 | | 240.142 | | 195.758 | |
| **Multilevel gamma regression** | | | | | | |
| Intercept | 5.0541 (0.4013) | <.0001*** | 3.4663 (0.7863) | <.0001*** | 3.7081 (0.8093) | <.0001*** |
| PANSE |  | | | | | |
| Q2 | 0.2729 (0.4915) | 0.5787 | 0.5186 (0.4910) | 0.2908 | 0.1081 (0.4962) | 0.8276 |
| Q3 | 1.2360 (0.4852) | 0.0108** | 1.3936 (0.4714) | 0.0031** | 0.7934 (0.4677) | 0.0898 |
| Q4 | 0.4798 (0.5309) | 0.3661 | 0.6555 (0.5172) | 0.2050 | -0.2444 (0.5219) | 0.6396 |
| Q5 | 0.2388 (0.4797) | 0.6185 | 0.4306 (0.4732) | 0.3628 | 0.1753 (0.4416) | 0.6914 |
| Age | - | - | 0.0252 (0.0111) | 0.0238 | 0.0147 (0.0113) | 0.1931 |
| Male | - | - | -0.4879 (0.4748) | 0.3042 | -0.6004 (0.4696) | 0.2010 |
| College | - | - | - | - | 1.2596 (0.4546) | 0.0056** |
| Some College | - | - | - | - | 1.7334 (0.5421) | 0.0014** |
| High Income | - | - | - | - | -0.6470 (0.3834) | 0.0916 |
| Low Income | - | - | - | - | -0.9257 (0.3468) | 0.0076** |
| **Fit Indices** | | | | | | |
| AIC | 706.7478 | | 705.6626 | | 634.5146 | |
| BIC | 718.5696 | | 721.4249 | | 656.9690 | |

Note: *significance at p<0.05, **significance at p<0.01 and ***significance at p<0.001

**Appendix Table 3: Estimates for Logistic: Weighted Moderate-to-vigorous Leisure time Physical Activity**

| Parameter | Unadjusted | | Model 1 | | Model 2 | |
| --- | --- | --- | --- | --- | --- | --- |
| **Multilevel logistic regression** | | | | | | |
|  | Estimate (SE) | P Value | Estimate (SE) | P Value | Estimate (SE) | P-Value |
| Intercept | -1.2528 (0.4629) | 0.0068 | -0.4515 (0.8404) | 0.5911 | -1.7407 (1.1278) | 0.1227 |
| PANSE (Ref: Q1) |  | | | | | |
| Q2 | 0.8279 (0.5582) | 0.1380 | 0.8441 (0.5617) | 0.1329 | 0.7369 (0.5000) | 0.1405 |
| Q3 | 1.3640 (0.5707) | 0.0169* | 1.3432 (0.5741) | 0.0193* | 1.0741 (0.5970) | 0.0720 |
| Q4 | 0.8473 (0.5643) | 0.1332 | 0.8811 (0.5676) | 0.1206 | 0.7660 (0.6167) | 0.2142 |
| Q5 | 1.7789 (0.5802) | 0.0022** | 1.7791 (0.5828) | 0.0023** | 1.5580 (0.6017) | 0.0096** |
| Age | - | - | -0.0146 (0.0129) | 0.2579 | -0.00535 (0.0144) | 0.7103 |
| Male | - | - | 0.0535 (0.5207) | 0.9182 | 0.0129 (0.5654) | 0.9818 |
| College | - | - | - | - | 0.8862 (0.6463) | 0.1703 |
| Some College | - | - | - | - | 0.6815 (0.6963) | 0.3278 |
| High Income | - | - | - | - | 0.2933 (0.4408) | 0.5058 |
| Low Income | - | - | - | - | 0.3397 (0.4055) | 0.4022 |
| Fit Indices |  | | | | | |
| AIC | 246.133 | | 248.837 | | 222.015 | |
| -2 Log L | 236.133 | | 234.837 | | 200.015 | |

Note: *significance at p<0.05, **significance at p<0.01 and ***significance at p<0.001

**Appendix Table 4: Estimates for Logistic and Gamma models: Muscle Strengthening Activity**

| Parameter | Unadjusted | | Model 1 | | Model 2 | |
| --- | --- | --- | --- | --- | --- | --- |
| **Multilevel logistic regression** | | | | | | |
|  | Estimate (SE) | P Value | Estimate (SE) | P Value | Estimate (SE) | P-Value |
| Intercept | -0.4700 (0.3291) | 0.1533 | -0.4257 (0.7522) | 0.5714 | -1.1554 (0.9671) | 0.2322 |
| PANSE (Ref: Q1) |  | | | | | |
| Q2 | 0.6836 (0.4409) | 0.1211 | 0.6599 (0.4446) | 0.1378 | 0.7369 (0.5000) | 0.1405 |
| Q3 | 1.0941 (0.4590) | 0.0171* | 1.0760 (0.4636) | 0.0203* | 1.3797 (0.5110) | 0.0069** |
| Q4 | 0.5610 (0.4466) | 0.2091 | 0.5516 (0.4473) | 0.2175 | 0.5978 (0.5158) | 0.2465 |
| Q5 | 1.2371 (0.4701) | 0.0085** | 1.2302 (0.4716) | 0.0091** | 1.4439 (0.5093) | 0.0046** |
| Age | - | - | -0.00094 (0.0114) | 0.9341 | 0.00369 (0.0131) | 0.7787 |
| Male | - | - | 0.2227 (0.4833) | 0.6449 | 0.3255 (0.5367) | 0.5442 |
| College | - | - | - | - | 0.0968 (0.5251) | 0.8538 |
| Some College | - | - | - | - | -0.0160 (0.5711) | 0.9776 |
| High Income | - | - | - | - | 0.2138 (0.4141) | 0.6056 |
| Low Income | - | - | - | - | 0.4083 (0.3761) | 0.2777 |
| Fit Indices |  | | | | | |
| AIC | 294.338 | | 298.120 | | 254.981 | |
| -2 Log L | 284.338 | | 284.120 | | 232.981 | |
| **Multilevel gamma regression** | | | | | | |
| Intercept | 1.0006 (0.1627) | <.0001*** | 0.7483 (0.3378) | 0.0267 | 0.2728 (0.3908) | 0.4851 |
| PANSE |  | | | | | |
| Q2 | -0.1140 (0.2044) | 0.5769 | -0.1354 (0.2054) | 0.5097 | -0.0187 (0.2264) | 0.9343 |
| Q3 | 0.0341 (0.2017) | 0.8658 | 0.0091 (0.2059) | 0.9647 | 0.2630 (0.2160) | 0.2233 |
| Q4 | -0.0887 (0.2092) | 0.6715 | -0.1135 (0.2096) | 0.5880 | 0.0732 (0.2369) | 0.7572 |
| Q5 | 0.1791 (0.2017) | 0.3744 | 0.1848 (0.2005) | 0.3568 | 0.3022 (0.2127) | 0.1553 |
| Age | - | - | 0.0041 (0.0048) | 0.3980 | 0.0057 (0.0048) | 0.2341 |
| Male | - | - | 0.2646 (0.1878) | 0.1588 | -0.0678 (0.1975) | 0.7316 |
| College | - | - | - | - | 0.0988 (0.2017) | 0.6244 |
| Some College | - | - | - | - | 0.4031 (0.2218) | 0.0691 |
| High Income | - | - | - | - | 0.4100 (0.1601) | 0.0105 |
| Low Income | - | - | - | - | -0.2007 (0.1450) | 0.1664 |
| **Fit Indices** | | | | | | |
| AIC | 448.7946 | | 449.9106 | | 351.5513 | |
| BIC | 465.5195 | | 472.2105 | | 382.4479 | |

Note: *significance at p<0.05, **significance at p<0.01 and ***significance at p<0.001
